# Thickness Optimization of Charge Transport Layers on Perovskite Solar Cells for Aerospace Applications

**DOI:** 10.3390/nano13121848

**Published:** 2023-06-13

**Authors:** Doowon Lee, Kyeong Heon Kim, Hee-Dong Kim

**Affiliations:** 1 Department of Semiconductor Systems Engineering, and Convergence Engineering for Intelligent Drone, Institute of Semiconductor and System IC, Sejong University, 209, Neungdong-ro, Gwangjin-gu, Seoul 05006, Republic of Korea; dwlee@sejong.ac.kr; 2 Department of Convergence Electronic Engineering, Gyeongsang National University, Jinju-si 52725, Republic of Korea; kkim@gnu.ac.kr

**Keywords:** solar cells, perovskite, aerospace, charge transport layer

## Abstract

In aerospace applications, SiO_x_ deposition on perovskite solar cells makes them more stable. However, the reflectance of the light changes and the current density decreases can lower the efficiency of the solar cell. The thickness of the perovskite material, ETL, and HTL must be re-optimized, and testing the number of cases experimentally takes a long time and costs a lot of money. In this paper, an OPAL2 simulation was used to find the thickness and material of ETL and HTL that reduces the amount of light reflected by the perovskite material in a perovskite solar cell with a silicon oxide film. In our simulations, we used an air/SiO_2_/AZO/transport layer/perovskite structure to find the ratio of incident light to the current density generated by the perovskite material and the thickness of the transport layer to maximize the current density. The results showed that when 7 nm of ZnS material was used for CH_3_NH_3_PbI_3_-nanocrystalline perovskite material, a high ratio of 95.3% was achieved. In the case of CsFAPbIBr with a band gap of 1.70 eV, a high ratio of 94.89% was shown when ZnS was used.

## 1. Introduction

Due to the harsh conditions in space, solar cells used in aerospace have to meet certain criteria, such as being highly efficient, resistant to radiation, lightweight, and reliable [1]. Researchers in the aerospace fields are interested in perovskite solar cells because they might be able to meet these needs. Perovskite solar cells are a new type of solar cell that have the potential to be highly efficient, lightweight, and thin. They also have the advantage of being relatively easy to manufacture, which can reduce costs [2,3,4]. Therefore, there is a lot of research being conducted on perovskite materials for solar cells [5,6,7,8]. In addition, perovskite solar cells have been shown to be more radiation-resistant than traditional silicon solar cells, which is a significant advantage for space applications [1,9]. Even though perovskite solar cells need to be made more reliable and efficient, they are one of the best ways to use solar power in space.

In order to improve their reliability for aerospace or space applications, the deposition of a silicon oxide (SiO_x_) layer on the surface of perovskite solar cells has been demonstrated by A. R. Kirmani et al. [10]. The research has shown that a 1 μm thick-SiO_x_ layer can significantly enhance the stability and reliability of perovskite solar cells for space applications. They reported that the deposited SiO_x_ layer blocks 0.05 MeV protons at a fluence of 10^15^ cm^−2^ without loss of efficiency losses, increasing device lifetime by a factor of 20 in low Earth orbit and by a factor of 30 in high elliptical orbit. In addition, the SiO_x_ layer acts as a barrier layer that prevents the ingress of moisture and the Na^+^ diffusion from the module front cover glass into the solar cell [11,12]. Moisture and Na^+^ can cause degradation of the solar cell, leading to a decrease in performance and the eventual failure of the solar cell. The SiO_x_ layer can act as a protective layer, increasing the lifetime of the perovskite solar cell and reducing the risk of failure due to moisture and Na^+^. The thick-SiO_x_ layer can reduce the formation of defects in the perovskite material, improving the overall performance and stability of the solar cell. Defects can cause the recombination of charge carriers, leading to the reduced efficiency and stability of the solar cell. The SiO_x_ layer can decrease the density of the defects in the perovskite material, reducing the recombination rate and improving the performance and stability of the solar cell [12]. While the deposition of a SiO_x_ layer can enhance the reliability and stability of perovskite solar cells for space applications, optimizing the thickness and material of each layer (such as the electron transport layer (ETL), the hole transport layer (HTL), transparent conducting oxide (TCO), and perovskite) is important for achieving high performance and reliability. Among them, the thickness of the ETL and HTL layers can affect the efficiency, stability, and reliability of the perovskite solar cell.

The thickness of the ETL and HTL layers has a particularly huge effect on the current density of the perovskite solar cell due to the anti-reflection coating (ARC) effect. An ARC is a thin layer deposited to the surface of the solar cell to minimize reflection losses and increase the amount of light that is absorbed by the perovskite material. The design of single-layer ARC is based on the principle of interference. When incident light comes to single-layer ARC with a refractive index different from that of air, a portion of the light is reflected back at the interface between the air and a perovskite material. Subsequently, incident light waves travel through single-layer ARC and meet the interface between single-layer ARC and the substrate, and a part of the light is reflected back. The interference between the wavelengths reflected between the interfaces of the air/single-layer ARC and the single-layer ARC/substrate determines the final reflectance. The thickness of the film is chosen so that the phase shift between the reflected and incident light waves is 180°. This means that the reflected light waves interfere destructively with the incident light waves, resulting in very low reflectance for a specific wavelength. The thickness of single-layer ARC film is chosen based on the refractive index of the material and the wavelength of the incident light. The optimal thickness of the ARC could be calculated using the formula [13]:

(1) $$t=\frac{\lambda }{4n}$$

where t is the thickness of the single-layer ARC, λ is the wavelength of the incident light, and n is the refractive index of the single-layer ARC material. The thickness of the single-layer ARC should be equal to one-quarter of the wavelength of the incident light divided by the refractive index of the single-layer ARC material. However, for perovskite solar cells, the structure of the front side commonly has Air/TCO/ETL/perovskite or Air/TCO/HTL/perovskite, which is equivalent to applying a double-layer ARC (DLARC) with TCO and ETL or TCO and HTL. DLARC is composed of two layers of materials with different refractive indices to minimize the reflection [14]. The principle behind the design of a DLARC is similar to that of a single-layer ARC. The two layers of the DLARC work together to reduce reflection because of the principle of optical interference. When incident light waves pass through the two layers, they interfere with each other in a way that reduces the amount of reflection. The thickness and refractive indices of the TCO and a charge transport layer (CTL) are thus carefully chosen to minimize reflection losses. However, although there have been many studies demonstrating the optimal thickness of the CTL layer to improve the current density and efficiency of solar cells in the absence of SiO_x_, in the case of perovskite solar cells for space applications, a thick layer of SiO_x_ is deposited on top of the perovskite layer requiring an additional optimization process. In order to minimize the reflectance in this case, it can be challenging to determine the optimal thickness and refractive index of ETL or HTL materials experimentally. This is because the interference effects between the multiple layers can become more complex and difficult to predict. Although this can be verified with some experimentation [15], it is time consuming and costly.

A simulation is a useful tool for designing a double-layer ARC for a solar cell because the design process is more complex than for a single-layer ARC. Simulation tools can help to model the behavior of light at the interface between the two layers, taking into account the thickness, refractive index, and absorption coefficient of each layer. By simulating the optical properties of different double-layer ARC designs, it is possible to identify the optimal combination of layer thicknesses and refractive indices to achieve the desired performance metrics, such as high light absorption and low reflection. However, there are not many studies via simulation for the perovskite solar cells with a barrier layer, and the appropriate CTLs for different perovskite materials have to be widely considered.

**Figure 1:**
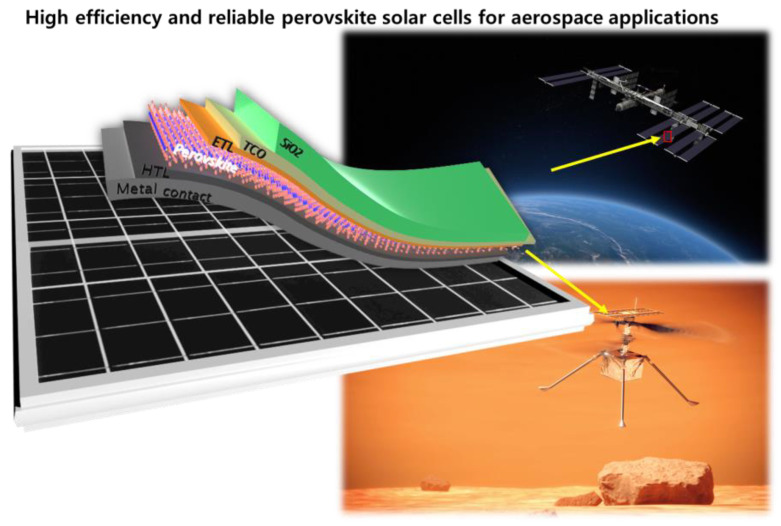
Schematic illustration of perovskite solar cells with SiO_x_ for aerospace applications.

This paper aims to provide a guide for selecting the ETL or HTL material that can maximize the current density of a perovskite solar cell depending on a perovskite material, as shown in Figure 1. The simulation was conducted by OPAL2 which is a useful tool to obtain the optimal thickness of ETL and HTL [16,17]. CH_3_NH_3_PbBr_3_-microcrystalline, CH_3_NH_3_PbI_3_-nanocrystalline, and CsFAPbIBr with different bandgaps were applied as perovskite materials. Simulations were performed to find the proper thickness of ETL and HTL for each material in order to minimize optical loss. Because of this, the optimal thickness and the ETL or HTL material that can be used to improve current density depend on the perovskite material. We observed that AlN, HfO_2_, SiN, WO_3_, ZnS, and TiO_2_ mostly improved the current density, while some perovskites were not suitable for TiO_2_ due to the high processing temperature. As a result, we have proposed materials and optimal thicknesses that may reduce reflections depending on perovskite materials, which is anticipated to support the development of perovskite solar cells or perovskite-based tandem solar cells for aerospace and space applications.

## 2. Materials and Methods

**Figure 2:**
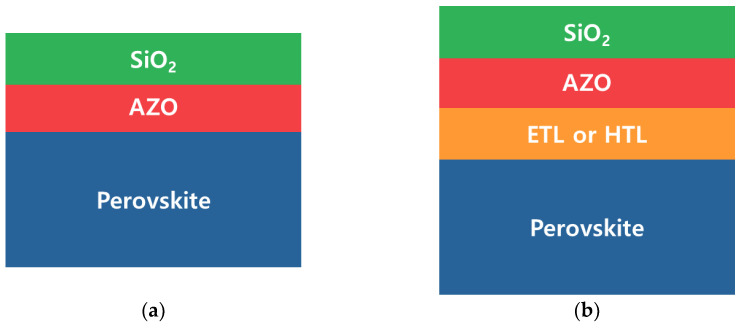
Schematic structure for the OPAL2 simulation. Reference structures (**a**) without ETL or HTL, (**b**) with ETL or HTL.

Using the OPAL 2 simulation tool, the experiment was performed. The surface morphology was set to random upright pyramids with a 54.74° angle and a 0% planar proportion. AM0 was employed as the incident illumination spectrum:

Figure 2 shows the schematic structure used in the simulation. The thickness of the top layer of SiO_x_ was decided to be 1 μm [10]. Thereafter, 80-nm AZO was used as the transparent electrode because AZO has better thermal stability in space than conventional ITO [18,19]. The thickness of the perovskite was set to 1 μm, which is the minimum thickness that can be applied in the simulation.

In order to precisely compare ETL and HTL materials for a perovskite material, we simply calculate the ratio with the equation below:

(2) $$\mathrm{Ratio}\;\left(\%\right)=\frac{\mathrm{Generated}\;\mathrm{current}\;\mathrm{density}\;\mathrm{in}\;a\;\mathrm{perovskite}\;\mathrm{material}}{\mathrm{Current}\;\mathrm{density}\;\mathrm{of}\;\mathrm{incident}\;\mathrm{light}}$$

The current density of incident light is the photon current density when all of the AM 1.5 sunlight is absorbed by the perovskite substrate. This ratio will be 100% if the substrate completely absorbs all of the sunlight. If there is reflection or absorption by the film, the current density generated by the perovskite material will be less than the current density of the incident light, and the ratio will fall below 100%. Because the current density of incident light varies with the perovskite material, we calculated the ratio. In order to find the proper ETL and HTL materials with optimal thickness for minimizing reflectance depending on the perovskite materials, we tried to use most of the materials available in the OPAL2 simulation. CH_3_NH_3_PbBR_3_ with microcrystalline structure, CH_3_NH_3_PBI_3_-nanocrystalline structure, Cs_0.05_(MA_0.166_FA_0.833_)_0.95_Pb(Br_0.166_I_0.833_)_3_, Cs_y_FA_1−y_Pb(I_x_Br_1−x_)_3_ with a band gap of 1.62, 1.67, 1.70, 1.73, 1.75, and 1.80 eV was applied as a perovskite material. AlN, Al_2_O_3_, CdS, Cu_2_ZnSnSe, HfO_2_, In_2_O_3_:H, InP, IZO, NiO, MgF_2_, SiC, SiN, SiO_x_N_y_, SiO_2_, SnO_2_, Spiro-OMeTAD, TiO_2_, V_2_O_x_, WO_3_, ZnO, and ZnS were used as an ETL or HTL material. The detailed information is shown in Table 1 and Table 2.

## 3. Results

**Figure 3:**
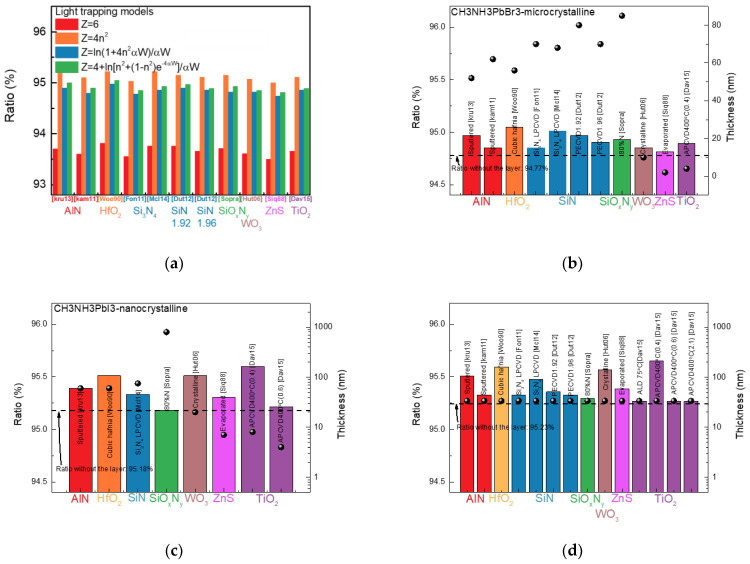
(**a**) Ratio based on light-trapping models. Simulation results for (**b**) CH_3_NH_3_PbBr_3_-microcrystalline, (**c**) CH_3_NH_3_PbI_3_-nanocrystalline, and (**d**) triple-cation perovskite materials. In the figures above, the dots represent the thickness of the optimized insulator that maximizes the ratio.

In order to assess the effects of the light-trapping models, simulations were performed using four different light-trapping models. CH_3_NH_3_PbBr_3_-microcrystalline perovskite material was used as a representative for the perovskite material. The light-trapping models were configured using the following formulas [24,25,26]:

(3) Z=6

(4) $$Z=4n^{2}$$

(5) $$Z=\frac{\ln\left(1+4n^{2}\alpha W\right)}{\alpha W}$$

(6) $$Z=4+\frac{\ln\left(n^{2}+\left(1–n^{2}\right)e^{-4\alpha W}\right)}{\alpha W}$$

where Z, n, α, and W represent the light trapping factor, the refractive index, the absorption coefficient, and the thickness of the perovskite. Figure 3a shows the ratio depending on light-trapping models. As a result, when the light-trapping model changed, AlN, HfO_2_, SiN, SiO_x_N_y_, WO_3_, ZnS, and TiO_2_ showed an improvement in the ratios for all models. Only the ratio exhibits small differences because the reflection and absorption in films change as the light-trapping model changes. The light-trapping model does not seem to have a significant impact on the selection of CTLs since the type of CTL does not change as the ratio changes. Therefore, for the rest of the simulation, we used the light-trapping model with the fewest assumptions. See Equation (6).

**Figure 4:**
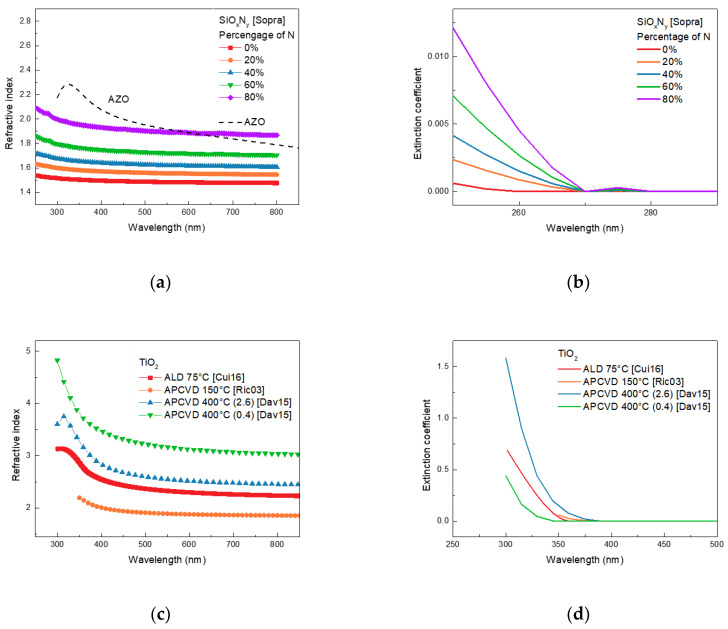
(**a**) the refractive index and (**b**) the extinction coefficient of SiO_x_N_y_ depending on the percentage of N. (**c**) the refractive index and (**d**) the extinction coefficient of TiO_2_ depending on the deposition condition and equipment.

Figure 3b,c show the simulation results for CH_3_NH_3_PbBr_3_ with microcrystalline and CH_3_NH_3_PbI_3_-nanocrystalline perovskite materials. The column graph displays the calculated ratio between the current density generated by a perovskite material and the incident current density. The black dots represent the optimal thickness of each material when the ratio is at its highest point. The black dashed line represents the ratio for the structure composed with 1 μm SiO_2_/80-nm AZO/perovskite materials as a reference ratio. As a result, out of 88 materials, only AlN, HfO_2_, SiN, SiO_x_N_y_, WO_3_, ZnS, and TiO_2_ show a higher ratio than the reference ratio. This indicates that these materials can increase their current density even after being inserted between an AZO and perovskite. In the case of other materials, the current density of the perovskite may decrease after the deposition of the material. In detail, in the case of CH_3_NH_3_PbBr_3_ with a microcrystalline structure, the highest ratio of 95.59% was obtained when 56-nm HfO_2_ (Cubic hafnia [Woo90]) was deposited on the perovskite, as shown in Figure 3b. Additionally, 68-nm SiN (Si_3_N_4_ LPCVD [Mcl14]), 80-nm SiN (PECVD1.92 [Dut12]), and 80-nm AlN (Sputtered [kru13]) showed high ratios of 95%, 94.97%, and 94.97%, respectively. Among them, Si_3_N_4_ exhibits the highest ratio due to its smaller difference in the refractive index with AZO than others and low extinction coefficient within the visible light wavelength range. The details are described later in Figure 4. HfO_2_ is an inert, wide bandgap, transparent metal oxide with a high dielectric constant and a large refractive index. It also has its own merits as a hole-transporting layer in polymer solar cells [27]. However, in perovskite solar cells, HfO_2_ is usually used as photoelectrodes and not as an HTL [28]. In addition, Si_3_N_4_, SiN, AlN, and WO3 are not frequently used as CTLs. TiO_2_, on the other hand, is commonly used as the ETL in perovskite solar cells, but its high operating temperature leads to the degradation and decomposition of perovskite solar cells during deposition [29,30]. As for ZnS, it is commonly used as an ETL [31], making it a suitable material for CH_3_NH_3_PbBr_3_.

In the case of CH_3_NH_3_PbI_3_ with a nanocrystalline structure, as shown in Figure 3c, the highest ratio of 96.51% was obtained using 60-nm HfO_2_ (Cubic hafnia [Woo90]), followed by 20-nm WO_3_ (Crystalline [Hut06]), 60-nm AlN (Sputtered [kru13]), 75-nm SiN (Si_3_N_4_ LPCVD [Mcl14]), 7-nm ZnS (Evaporated [Siq88]), and 8-nm TiO_2_ (APCVD400 °C (0.4) [Dav15]), which all showed high ratios of 95.6% or higher. In the case of SiO_x_N_y_ (80%N [Sopra]), the optimal condition requires its thickness to exceed the upper bound of OPAL2. Although SiO_x_N_y_ is not perfectly optimized, we believe that it could still improve or maintain the ratio without losing reflected light. However, as we mentioned earlier, ZnS is more suitable as an ETL for CH_3_NH_3_PbI_3_ with a nanocrystalline structure. Therefore, ZnS is a great candidate to minimize reflectance loss and maximize current density in CH_3_NH_3_PbI_3_-based solar cells.

Figure 3d illustrates the simulation results for the triple-cation perovskite material. Interestingly, similar to the CH_3_NH_3_PbBr_3_ and CH_3_NH_3_PbI_3_ material, the triple-cation perovskite material also exhibits high ratios when AlN, HfO_2_, SiN, SiO_x_N_y_, WO_3_, ZnS, and TiO_2_ are deposited. The highest ratio of 95.65% was observed when 33.59-nm TiO_2_ was deposited using APCVD with an H_2_O/tetra isopropyl titanate ratio of 0.4, followed by HfO_2_ and WO_3_ with a crystalline structure having the highest ratio of 95.56%. As mentioned earlier, more research is required to use AlN, HfO_2_, SiN, SiO_x_N_y_, and WO_3_ as a CTL. TiO_2_ can be used since the TiO_2_ deposited by ALD at 75 °C shows an improved ratio. Therefore, ZnS and TiO_2_ can be excellent candidates for an ETL.

To further analyze the correlation between the above ratios and materials, we analyzed the refractive index and extinction coefficient of SiO_x_N_y_ and TiO_2_. Figure 4a,b show the refractive index and extinction coefficient of SiO_x_N_y_ depending on the N contents. It was observed that the refractive index and extinction coefficient increased with increasing N content. The reason for the increase in the ratio for SiO_x_N_y_ with 80% N despite the increase in the extinction coefficient seems to be that the refractive index of SiO_x_N_y_ has the smallest difference from that of AZO, which reduces reflectance. Figure 4c,d show the refractive index and extinction coefficient of TiO_2_ materials depending on deposition conditions and equipment. In the above result, it was observed that APCVD 400 °C (0.4) [Dav15] can improve the ratio for all perovskite materials. However, other TiO_2_ materials were unable to increase the ratio despite showing a small difference in the refractive index between AZO. This is because it shows a higher extinction coefficient than APCVD 400 °C (0.4) [Dav15], as shown in Figure 4d, resulting in high absorption in film. Therefore, to minimize reflectance, the refractive index is important, but the extinction coefficient must also be considered.

To determine the cause of the variation in the ratio, we compared the percentage of light reflected and absorbed by the film for the CH_3_NH_3_PbBr_3_ microcrystalline perovskite material, which showed high improvements in the ratio for most CTL materials. AlN (Sputtered [kru13]), HfO_2_ (Cubic hafnia [Woo90]), SiN (Si_3_N_4_ LPCVD [Mcl14]), SiO_x_N_y_ (80%N [Sopra]), WO_3_ (Crystalline [Hut06]), ZnS (Evaporated [Siq88]), and TiO_2_ (APCVD400 °C (0.4) [Dav15]), which showed the high improvement in current for each material, were selected to analyze the ratio of light reflected and the ratio of light absorbed in the films. In order to analyze the ratio of reflected light, we analyzed it in the wavelength range from 390 to 610 nm, where the intensity of incident light is strongest, because there were no obvious trends when comparing across all wavelengths and materials.

**Figure 5:**
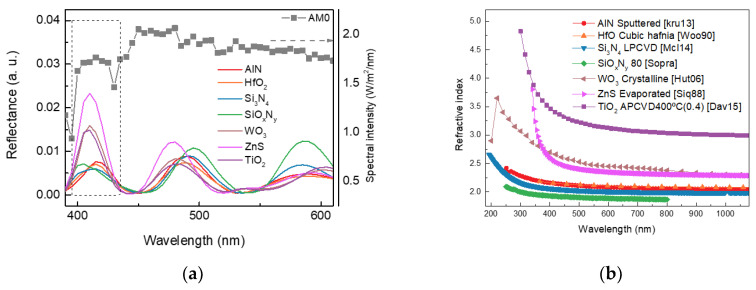

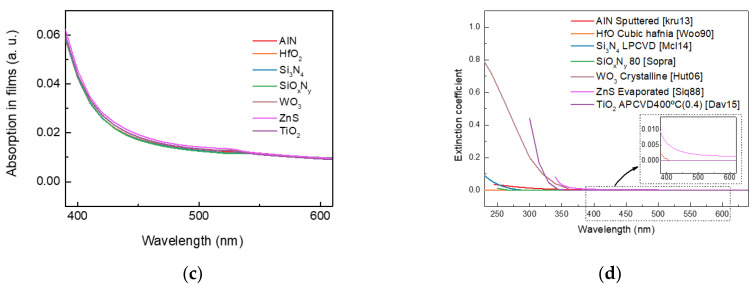
In the structure of the SiO_x_/AZO/CTL for the CH_3_NH_3_PbBr_3_-microcrystalline perovskite material, the ratio of (**a**) reflectance, (**b**) the refractive index of CTL materials, (**c**) absorption in films, and (**d**) extinction coefficient of CTL materials where the enlarged figure depicts the extinction coefficient in the wavelength range from 390 to 610 nm.

Figure 5a shows the ratio of the reflected light for the SiO_2_/AZO/CTL structure with each CTL material in the wavelength range from 390 to 610 nm. The black line shows the spectral intensity of the incident light as a function of wavelength. The other colored lines represent the ratio of reflected light in the structure of the SiO_2_/AZO/CTL when a CTL with an optimized thickness that minimizes the reflectance is applied. Although we could not observe a significant change trend in the 390 and 610 wavelengths, we could observe a specific trend in the wavelength range from ~395 to 440 nm, which is the wavelength range of the squares with black dash lines in Figure 5a. For CTLs, such as WO_3_, ZnS, and TiO_2_ showing a less improved ratio, the reflectance in the wavelength range is high above 0.01. Other CTLs with a better-enhanced ratio show low reflectance in the wavelength range below 0.01. This change is due to the large reflective indices of WO_3_, ZnS, and TiO_2_ at these wavelengths, as shown in Figure 5b. In addition, it can be observed that the amount of light absorbed by the film does not change significantly with wavelength and material, as shown in Figure 5c. Even when 85-nm SiO_x_ is used as CTL, the ratio of absorbed light exhibits only minor changes. These trends of reflectance and absorption are similarly represented for other CTLs and perovskite materials. In the above results, the reason why the reflectance at that wavelength range has a significant impact on the ratio is that perovskite solar cells typically have high external quantum efficiency at that wavelength, and the spectral intensity of the solar cell is large [32,33]. In addition, the extinction coefficients of the CTL are low in that wavelength range, as shown in Figure 5d. Although the extinction coefficient becomes larger at the lower wavelength range for WO_3_, the effect seems to be minor since sunlight at the wavelength range is expected to be absorbed earlier by the upper thick SiO_2_. Therefore, in the SiO_2_/AZO/CTL structure, materials that can reduce the reflectance from 395 to 440 wavelengths are expected to show better current density.

**Figure 6:**
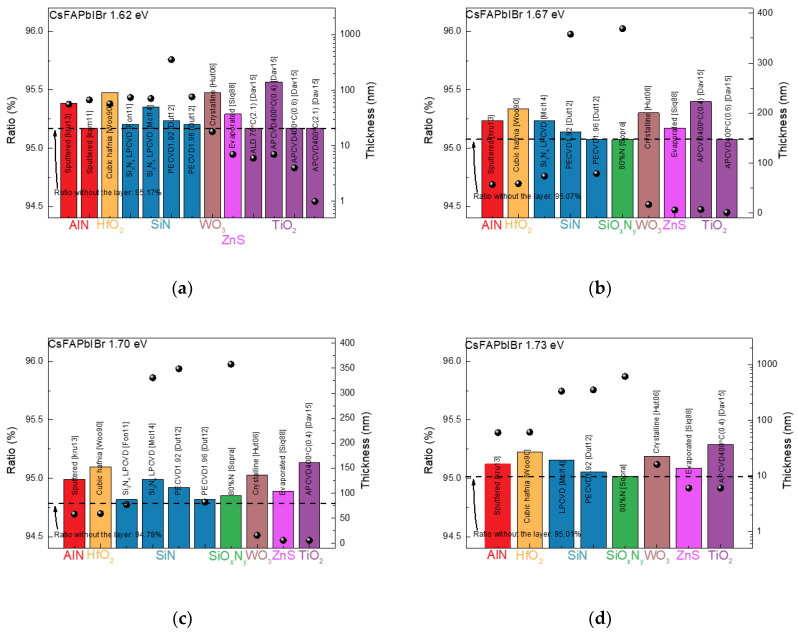

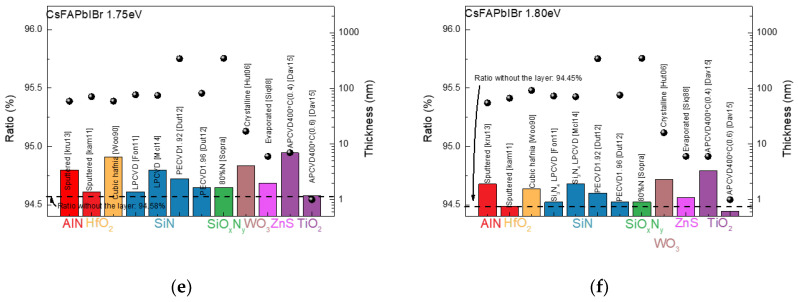
Simulation results for CsFAPbIBr perovskite material with a bandgap of (**a**) 1.62, (**b**) 1.67, (**c**) 1.70, (**d**) 1.73, (**e**) 1.75, and (**f**) 1.80 eV. In the figures above, the dots show the thickness of the optimized insulator that maximizes the ratio.

Figure 6 shows the simulation results for cesium-formamidinium-based mixed-halide (CsFAPbIBr) with band gaps ranging from 1.62 to 1.80 eV. For the band gap of 1.62 eV, AlN, HfO_2_, SiN, WO_3_, ZnS, and TiO_2_ show improved ratios compared to the reference, as shown in Figure 6a. AlN (Sputtered [kru13]), HfO_2_ (Cubic hafnia [Woo90]), SiN (Si_3_N_4_ LPCVD [Mcl14]), WO_3_ (Crystalline [Hut06]), and TiO_2_ (APCVD400 °C (0.4) [Dav15]) showed noticeable improvements. However, as mentioned above, these materials still require a lot of research. ZnS (Evaporated [Siq88]) showed an improved ratio of 0.22% over the reference and is thus considered an excellent ETL material.

Figure 6b shows the simulation results for CsFAPbIBr with a band gap of 1.67 eV. AlN, HfO_2_, SiN, SiO_x_N_y_, WO_3_, ZnS, and TiO_2_ exhibit improved ratios. TiO_2_ (APCVD400 °C (0.4) [Dav15]) shows the highest ratio of 95.4% with a thickness of 7 nm. Moreover, 59-nm HfO_2_ (Cubic hafnia [Woo90]) and 17-nm WO_3_ (Crystalline [Hut06]) show ratios over 93%. In addition, 74-nm SiN (Si_3_N_4_ LPCVD [Mcl14]) and 57-nm AlN (Sputtered [kru13]) exhibit ratios above 92%. Finally, 6-nm ZnS (Evaporated [Siq88]) reveals a high ratio of 95.17%. Although TiO_2_, HfO_2_, WO_3_, and SiN show obviously higher ratios than others, their other characteristics as ETLs should be studied. Therefore, 6-nm ZnS is suitable for the CsFAPbIBr with a band gap of 1.67 eV.

For 1.70-eV CsFAPbIBr, as shown in Figure 6c, AlN, HfO_2_, SiN, SiO_x_N_y_, WO_3_, ZnS, and TiO_2_ showed enhanced ratios compared to the reference. TiO_2_ (APCVD400 °C (0.4) [Dav14]) with a thickness of 70 nm showed the highest ratio of 95.29%. Moreover, 61-nm HfO_2_ (Cubic hafnia [Woo90]), 16-nm WO_3_ (Crystalline [Hut06]), and 60-nm AlN (Sputtered [kru13]) showed improved ratios of 95.22, 95.19, and 95.12%, respectively. In the case of SiN (Si_3_N_4_ LPCVD [Mcl14]), the optimized thickness and ratio were 335 nm and 95.15%. The most suitable material, ZnS (Evaporated [Sqi88]), showed a relatively low ratio of 99% at a thickness of 12 nm.

In the case of CsFAPbIBr with a band gap of 1.73 eV, similar to the previous materials, AlN, HfO_2_, SiN, SiO, SiO_x_N_y_, WO_3_, ZnS, and TiO_2_ exhibited improved ratios, as shown in Figure 6d. The structure with 6-nm TiO2 (APCVD400 °C (0.4) [Dav14]) showed the highest ratio of 95.29%. High ratios above 95.1% were also observed for 60-nm AlN (Sputtered [kru13]), 61-nm HfO_2_ (Cubic hafnia [Woo90]), 335-nm SiN (Si3N4 LPCVD [Mcl14]), and 16-nm WO_3_ (Crystalline [Hut06]). Additionally, 6-nm ZnS exhibited a high ratio of 95.09%.

Figure 6e,f show the simulation results for CsFAPbIBr with band gaps of 1.75 and 1.80 eV. AlN, HfO_2_, SiN, SiO, SiO_x_N_y_, WO_3_, ZnS, and TiO_2_ also exhibited increased ratios. TiO_2_ (APCVD400 °C (0.4) [Dav14]) showed the highest ratio for both perovskite materials. AlN, HfO_2_, SiN, WO_3_, and ZnS exhibited noticeable improvement in the ratio as well. Therefore, CsFAPbIBr materials showed improved ratio values for AlN, HfO_2_, SiN, WO_3_, ZnS, and TiO_2_, except for the one with a bandgap of 1.62 eV. For a perovskite material with a bandgap of 1.62 eV, SiO_x_N_y_ did not show any improvement in the ratio. In addition, TiO_2_ (APCVD400 °C (0.4) [Dav14]), AlN (Sputtered [kru13]), HfO_2_ (Cubic hafnia [Woo90]), SiN (Si_3_N_4_ LPCVD [Mcl14]), WO_3_ (Crystalline [Hut06]), and ZnS (Evaporated [Sqi88]) exhibited improved ratios for all CsFAPbIBr perovskite materials. Since TiO_2_ requires a high-temperature process of more than 400 °C to obtain an increased ratio, studies are essential to obtain similar optical properties at lower processing temperatures for space perovskite solar cells. For AlN, HfO_2_, SiN, and WO_3_, there are not many studies on these materials as ETL or HTL for perovskite solar cells, so additional studies are needed to apply them to perovskite solar cells. We thus believe that ZnS is the best candidate for space-grade perovskite solar cells due to its excellent ETL properties and optical characteristics.

## 4. Conclusions

In this paper, our aim was to find the proper thickness and materials that can minimize optical losses when applied as ETL or HTL to space-grade perovskite solar cell structures through simulation. As a result, among the various materials, AlN, HfO_2_, SiN, SiO_x_N_y_, WO_3_, ZnS, and TiO_2_ were found to increase the ratio for converting the light from the incident light compared to structures without ETL or HTL. In addition, in the wavelength range from 395 to 470 nm, we observed that the ratio increases with lower reflectance. For CH_3_NH_3_PbI_3_ with a nanocrystalline structure, 8-nm TiO_2_ showed the highest ratio of 95.6%. However, it is difficult to use as ETL due to the high processing temperatures. Furthermore, additional research is required for AlN, HfO_2_, SiN, SiO_x_N_y_, and WO_3_ before they can be applied as ETLs and HTLs for perovskite solar cells. Therefore, it appears that ZnS is the most suitable material for ETLs in space perovskite solar cells. This paper serves as basic research on perovskite solar cells for aerospace applications as it presents properties that may enhance the absorption of light in a variety of ETL, HTL, and perovskite materials.

## Figures and Tables

**Table 1:** Types and variable names of perovskite materials used in the simulation.

Perovskite Materials	Variation Names	Reference
CH_3_NH_3_PbBr_3_	CH_3_NH_3_PbBr_3_-microcrystalline	[20]
CH_3_NH_3_PbI_3_	CH_3_NH_3_PbI_3_-nanocrystalline	[21]
Cs_0.05_(MA_0.166_FA_0.833_)_0.95_Pb(Br_0.166_I_0.833_)_3_	Triple-cation	[22]
Cs_y_FA_1-y_Pb(I_x_Br_1−x_)_3_	1.62 eV	[23]
	1.67 eV	
	1.70 eV	
	1.73 eV	
	1.75 eV	
	1.80 eV	

**Table 2:** Types and variable names of ETL and HTL materials used in the simulation.

ETL or HTL			
Materials	Variation Names	Materials	Variation Names
AlN	AlN Sputtered [kru13]	TiO_2_	ALD 750 °C [Cui16]
	AlN Sputtered [kam11]		APCVD 150 °C [Ric03]
Al_2_O_3_	Al_2_O_3_ on glass [Kum09]		APCVD 200 °C [Ric03]
	Al_2_O_3_ on Si [Kim97]		APCVD 250 °C [Ric03]
	Al_2_O_3_ on Si [Kum09]		APCVD 250 °C (0.4) [Dav15]
	Al_2_O_3_ on SiO_2_ [Kim97]		APCVD 250 °C (1.0) [Dav15]
CdS	[EIA15]		APCVD 250 °C (1.6) [Dav15]
Cu_2_ZnSnSe	Sputtered [EIA15]		APCVD 250 °C (3.1) [Dav15]
HfO_2_	Cubic Hafnia [Woo90]		APCVD 250 °C (4.2) [Dav15]
In_2_O_3_:H	ALD, amorphous [Mac14]		APCVD 250 °C (5.2) [Dav15]
	ALD, crystallized [Mac14]		APCVD 250 °C (8.4) [Dav15]
InP	Cubic [Pal85i]		APCVD300 °C [Ric03]
IZO	Amorphous, annealed [Mor15]		APCVD350 °C [Ric03]
NiO	ALD[Kou19]		APCVD400 °C [Ric03]
MgF_2_	Evaporated [Siq88]		APCVD400 °C [Tho08]
	single crystal-e		APCVD400 °C (0.4) [Dav15]
	single crystal-o		APCVD400 °C (0.6) [Dav15]
SiC	PECVD AK400 [Ste10]		APCVD400 °C (0.8) [Dav15]
	PECVD SiNA [Ste10]		APCVD400 °C (1.0) [Dav15]
SiN	Si_3_N_4_ LPCVD [Fon11]		APCVD400 °C (1.3) [Dav15]
	Si_3_N_4_ LPCVD [McI14]		APCVD400 °C (2.1) [Dav15]
	PECVD [Bak11]		APCVD400 °C (2.6) [Dav15]
	PECVD1.91 [Vog15]		APCVD400 °C (3.1) [Dav15]
	PECVD1.92 [Dut12]		APCVD400 °C (4.2) [Dav15]
	PECVD1.96 [Dut12]		APCVD450 °C [Ric03]
	PECVD1.99 [Dut12]		APCVD annealed 1000 °C [Ric03]
	PECVD2.03 [Dut12]		APCVD annealed 450 °C [Ric03]
	PECVD2.09 [Vog15]		APCVD annealed 500 °C [Ric03]
	PECVD2.13 [Vog15]		APCVD annealed 600 °C [Ric03]
	PECVD2.15 [Dut12]		APCVD annealed 700 °C [Ric03]
	PECVD2.37 [Dut12]		APCVD annealed 800 °C [Ric03]
	PECVD2.61 [Dut12]		APCVD annealed 900 °C [Ric03]
	PECVD2.71 [Dut12]		Mesoporous [Rao19]
SiO_x_N_y_	0%N [Sopra]		Spray [Ric00]
	20%N [Sopra]	V_2_O_x_	Thermal evaporation [Nas21]
	40%N [Sopra]	WO_3_	amorphous [Hut06]
	60%N [Sopra]		crystalline [Hut06]
	80%N [Sopra]	ZnO	LPCVD B-doped [Fan15]
SiO_2_	[Rao19]		LPCVD low k [Hol1 2b]
	Thermal [Pal85e]		Sputtered [ElA15]
SnO_2_	Fluor-doped [Rao19]	ZnS -	Evaporated [Siq88]
	Undoped [Rao19]		Hexagonal-e [Pal85h]
Spiro-OMeTAD	[Fil15]		Hexagonal-o [Pal85h]
	[Rao19]		-

## Data Availability

The data presented in this study are available on request from the corresponding author.
